# New Provisional Function of OmpA from *Acinetobacter* sp. Strain SA01 Based on Environmental Challenges

**DOI:** 10.1128/mSystems.01175-20

**Published:** 2021-01-12

**Authors:** Shahab Shahryari, Mahbubeh Talaee, Kamahldin Haghbeen, Lorenz Adrian, Hojatollah Vali, Hossein Shahbani Zahiri, Kambiz Akbari Noghabi

**Affiliations:** a Department of Energy & Environmental Biotechnology, National Institute of Genetic Engineering and Biotechnology (NIGEB), Tehran, Iran; b Department Environmental Biotechnology, Helmholtz Centre for Environmental Research-UFZ, Leipzig, Germany; c Chair for Geobiotechnology, Technische Universität Berlin, Berlin, Germany; d Department of Anatomy and Cell Biology, McGill University, Montreal, Quebec, Canada; University of California San Diego

**Keywords:** *Acinetobacter* sp. SA01, phenol, OmpA, emulsifying ability, porin, oxidative stress, outer membrane vesicle (OMV)

## Abstract

An outer membrane protein A (OmpA) from *Acinetobacter* sp. strain SA01 was identified and characterized in-depth based on the structural and functional characteristics already known of its homologues. *In silico* structural studies showed that this protein can be a slow porin, binds to peptidoglycan, and exhibits emulsifying properties. Characterization of the recombinant SA01-OmpA, based on its emulsifying properties, represented its promising potentials in biotechnology. Also, the presence of SA01-OmpA in outer membrane vesicles (OMV) and biofilm showed that this protein, like its homologues in Acinetobacter baumannii, can be secreted into the extracellular environment through OMVs and play a role in the formation of biofilm. After ensuring the correct selection of the protein of interest, the role of oxidative stress induced by cell nutritional parameters (utilization of specific carbon sources) on the expression level of OmpA was carefully studied. For this purpose, the oxidative stress level of SA01 cell cultures in the presence of three nonrelevant carbon sources (sodium acetate, ethanol, and phenol) was examined under each condition. High expression of SA01-OmpA in ethanol- and phenol-fed cells with higher levels of oxidative stress than acetate suggested that oxidative stress could be a substantial factor in the regulation of SA01-OmpA expression. The significant association of SA01-OmpA expression with the levels of oxidative stress induced by cadmium and H_2_O_2_, with oxidative stress-inducing properties and lack of nutritional value, confirmed that the cells tend to harness their capacities with a possible increase in OmpA production. Collectively, this study suggests a homeostasis role for OmpA in *Acinetobacter* sp. SA01 under oxidative stress besides assuming many other roles hitherto attributed to this protein.

**IMPORTANCE** *Acinetobacter* OmpA is known as a multifaceted protein with multiple functions, including emulsifying properties. Bioemulsifiers are surface-active compounds that can disperse hydrophobic compounds in water and help increase the bioavailability of hydrophobic hydrocarbons to be used by degrading microorganisms. In this study, an OmpA from *Acinetobacter* sp. SA01 was identified and introduced as an emulsifier with a higher emulsifying capacity than Pseudomonas aeruginosa rhamnolipid. We also showed that the expression of this protein is not dependent on the nutritional requirements but is more influenced by the oxidative stress caused by stressors. This finding, along with the structural role of this protein as a slow porin or its role in OMV biogenesis and biofilm formation, suggests that this protein can play an important role in maintaining cellular homeostasis under oxidative stress conditions. Altogether, the present study provides a new perspective on the functional performance of *Acinetobacter* OmpA, which can be used both to optimize its production as an emulsifier and a target in the treatment of multidrug-resistant strains.

## Abstract (precis)

*Acinetobacter* OmpA is known as a multifaceted protein with multiple functions, including emulsifying properties. Bioemulsifiers are surface-active compounds that can disperse hydrophobic compounds in water and help increase the bioavailability of hydrophobic hydrocarbons to be used by degrading microorganisms.

## INTRODUCTION

The genus *Acinetobacter*, as Gram-negative bacteria being in the class of *Gammaproteobacteria*, includes a variety of clinical and environmental species (1–3). The most important clinically relevant species, such as Acinetobacter baumannii, are usually multidrug resistant and can trigger several diseases (4). Also, a variety of potential biotechnological applications have been proposed for environmental strains of *Acinetobacter*, such as bioremediation of environmental pollutants or the production of emulsifying agents with the capability of stabilizing oil particles in water (5–8). One of the emulsifiers produced by Acinetobacter radioresistens strain KA53 is an OmpA-like protein with 349 amino acids, called AlnA, which shares clear sequence similarity with OmpA of Escherichia coli and OprF, the major porin of Pseudomonas aeruginosa (9, 10). OmpA from other oil-degrading members of *Acinetobacter*, such as *Acinetobacter* sp. strain ADP1, also possess emulsifying ability (11). The secretion of the OmpA, with emulsifying ability, to the extracellular environment of these *Acinetobacter* strains suggested that this protein is a secretory protein to increase the bioavailability of hydrophobic substrates with nutritional values. On the other hand, it has been shown that A. baumannii OmpA, as a porin, can be secreted into the surrounding environment via outer membrane vesicles (OMVs) (12–14). Also, OmpA of A. baumannii plays an important role in the modulation of the biogenesis of OMVs and biofilm formation (15, 16), contributing to bacterial survival under stress conditions (17, 18). In addition, different factors have been shown to stimulate the expression of OmpA, for example, the presence of ethanol or iron repletion in cultures of A. baumannii strains ATCC 17978 and A. baumannii ATCC 19606, respectively (19–21). A. radioresistens S13, a nonclinical strain, produced more OmpA during the stationary phase of growth than in the exponential phase (22, 23). Also, the expression of OmpA was higher in cells fed with phenol or benzoate than in cells fed with acetate (22). It was concluded that the increase in expression of OmpA could be mostly due to the hydrophobicity of phenol and benzoate, enhancing the bioavailability of hydrophobic carbon substrates.

Some surface-active compounds like rhamnolipids are produced by different strains of *Pseudomonas* and *Burkholderia* for various purposes, including quorum sensing, cell motility, and stimulation of hydrophobic compounds uptake by bacteria (24–26). The production of rhamnolipid can be affected by several factors, including the type of carbon source (27) or oxidative stress (28). According to published reports concerning the overexpression of OmpA in *Acinetobacter* strains, a protein with emulsifying ability, it would appear that the favorable conditions to elevate the level of stress in the cells are of immense importance. Foremost among them, the induction of oxidative stress and its potential effects on the expression of *Acinetobacter* OmpA, appear to be worth studying (Table 1).

**TABLE 1:** Conditions which, although used by cells, are considered a stimulator of OmpA expression in different *Acinetobacter* strains

Factors that influence the production of OmpA	Possible mechanism of oxidative stress	Reference no.
Iron repletion	Free radicals produced by iron Fenton reaction	91
Ethanol exposure	Fenton reaction as a result of iron released from damaged Fe-S proteins and, consequently, production of free radicals	38, 92
Phenol and benzoate exposure	Potential pro-oxidant under some circumstances, such as high concn and presence of metal ions or through degradation	93 – 95
Stationary phase	Possibly free radical production by essential nutrient starvations and/or normal metabolic changes within cells	96 – 98

In this study, we aimed to evaluate the importance of the nature of carbon source and oxidative stress induction that resulted from it on OmpA gene expression in a newly characterized phenol-degrading *Acinetobacter* sp. strain SA01 to provide a more in-depth insight concerning the OmpA function in *Acinetobacter* spp. (29). The genomic diversity among *Acinetobacter* strains and their adaptive evolution can cause physiological variations among them (30). Moreover, there are different variants of OmpA (with potentially different physicochemical properties) in the *Acinetobacter* population, which might be due to the lateral gene transfer (31). Therefore, the entire genome of *Acinetobacter* sp. SA01 was sequenced, and a series of *in silico* studies was performed to precisely identify the candidate genes responsible for encoding OmpA and stress response proteins. To further ensure the selection of OmpA among the candidates, its emulsifying performance, secretion into the extracellular environment via OMV, and its presence in biofilm were thoroughly examined. Then, the association of OmpA expression with oxidative stress, as induced by carbon source, was evaluated in the cells grown in sodium acetate, ethanol, and phenol as the sole carbon source. Additional experiments were performed to validate the possible relationship between OmpA expression and oxidative stress by employing cadmium and H_2_O_2_ as two oxidative stress-inducing agents.

## RESULTS

### Phylogenetic relationship based on whole proteome and identification of candidate OmpA.

**FIG 1:**
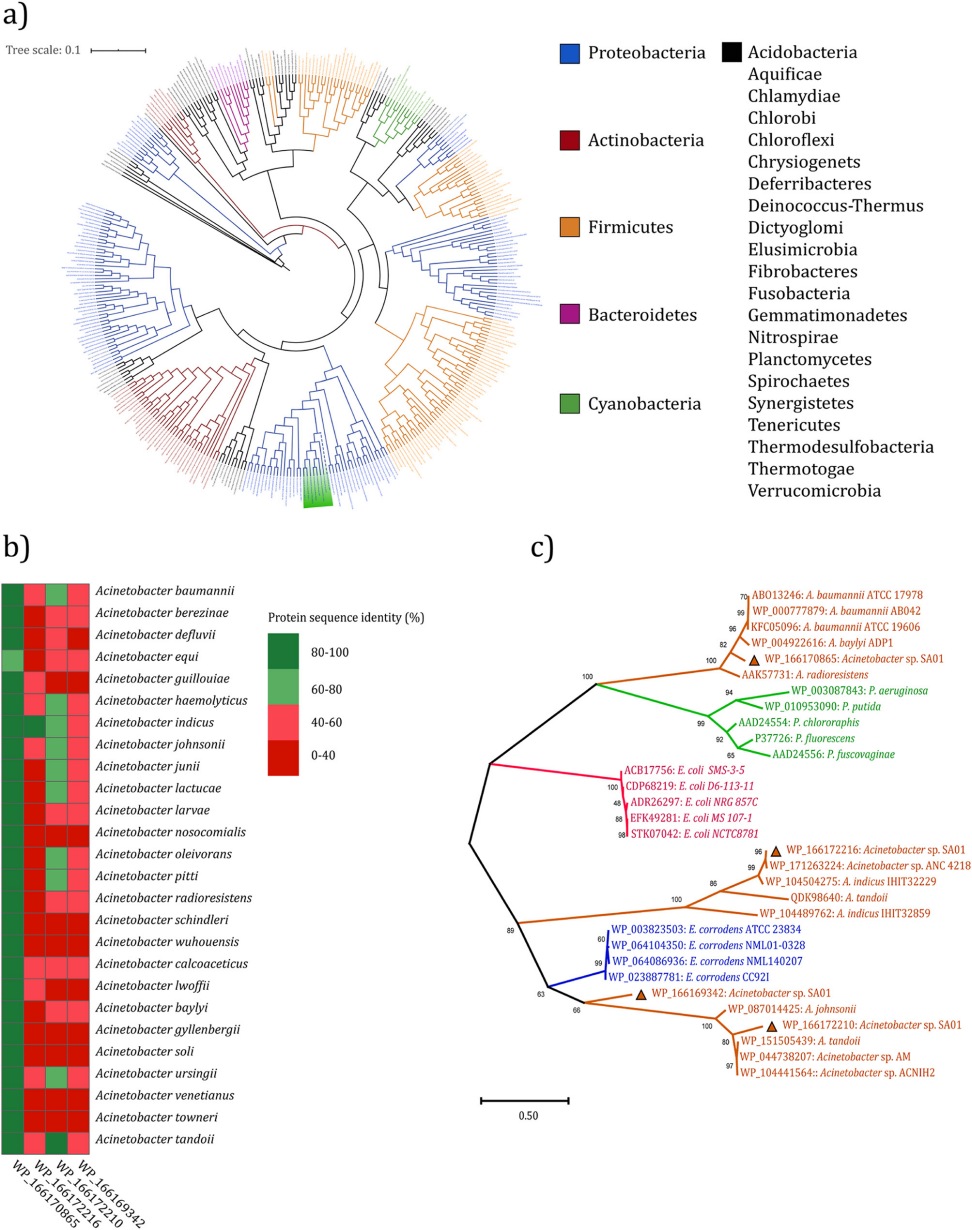
(a) Phylogenetic tree of *Acinetobacter* sp. SA01 based on the whole proteome. The genus *Acinetobacter* is highlighted green, and the dashed node indicates *Acinetobacter* sp. SA01. (b) Sequence identity score of 4 OmpA family proteins identified in *Acinetobacter* sp. SA01 with proteins of different species of *Acinetobacter*. (c) Evolutionary analysis of 4 OmpA family proteins identified in *Acinetobacter* sp. SA01, as labeled by triangle. Homolog proteins from *Acinetobacter* genus, orange; *Pseudomonas* genus, green; E. coli, pink; Eikenella corrodens, blue.

The assembly of DNA fragments obtained from next-generation shotgun sequencing resulted into 68 scaffolds and total sequence length of 3,451,566 bp. Around 3,357 protein-coding sequences were predicted by Prokka v1.10 (Data Sets S1 and S2 at https://doi.org/10.6084/m9.figshare.13415789). Phylogenetic tree based on total proteome confirmed that the SA01 strain belongs to the genus *Acinetobacter* (Fig. 1a). Whole-genome BLAST showed that the strain Acinetobacter kookii ANC 4667 with 79% query coverage and 90.15% sequence identity is the closest strain to *Acinetobacter* sp. SA01. Our genome survey showed that the *Acinetobacter* sp. SA01 annotated genome sequence includes loci from four genes encoding OmpA family proteins (GenPept accession nos. WP_166170865, WP_166172216, WP_166172210, and WP_166169342) with 351, 265, 284, and 180 amino acids, respectively. Among them, the annotated protein referred by the WP_166170865 accession number had the highest amino acid identity (over 80%) to the OmpAs of 25 *Acinetobacter* species, including *Acinetobacter* sp. ADP1 (WP_004922616), A. radioresistens KA53 (AAK57731), and A. baumannii ATCC 17978 (ABO13246) and selected for further analysis (Fig. 1b and c and Fig. S1 at https://doi.org/10.6084/m9.figshare.13415789).

### Bioinformatic analysis of candidate OmpA.

**FIG 2:**
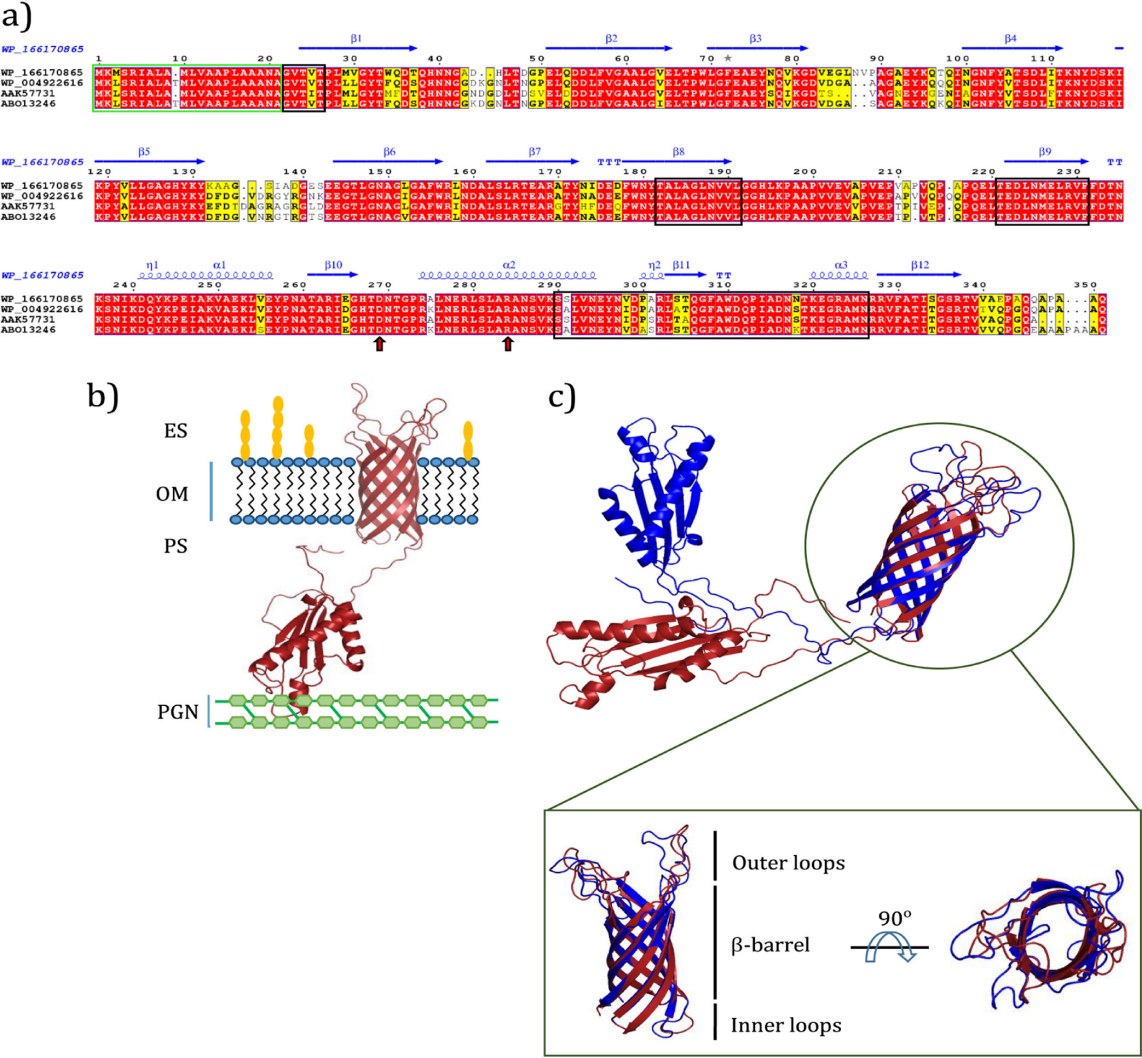
*In silico* studies of OmpA in *Acinetobacter* sp. SA01. (a) Multiple sequence alignment of OmpA. Sequences enclosed by green box demonstrate signal peptides. The sequences, in black box, display important regions that involved in emulsifying ability. Residues pointed by arrows are associated with anchoring of OmpA to peptidoglycan. Secondary structure information of each sequence is represented above it. (b) Predicted three-dimensional structure of OmpA and its orientation in the outer membrane (OM). Outer loops placed in extracellular space (ES) and OmpA-like domain placed in periplasmic space (PS) and binds to peptidoglycan (PGN). (c) Multiple structure alignment of OmpA β-barrel in *Acinetobacter* sp. SA01 and A. baumannii 17978.

The annotated protein referred to as WP_166170865 consists of three distinct domains, including an N-terminal signal peptide, a β-barrel, and an OmpA-like domain at the C terminus. The β-barrel domain of SA01-OmpA, like its homologs in other *Acinetobacter* (31), consists of eight β-strands connected with four periplasmic inner loops and four outer membrane loops (Fig. 2a). The OmpA-like domain starts from amino acid 219 and ends at amino acid 337, which is remarkably conserved compared to the OmpA amino acid sequence of the other three strains (Fig. 2a and Fig. S2 at https://doi.org/10.6084/m9.figshare.13415789). These include the presence of Asp271 and Arg286 as involved in the binding of OmpA to diaminopimelate (DAP)-peptidoglycan (PGN) and anchoring to the bacterial cell wall (32). Also, four regions, being important in the emulsifying capacity of AlnA, were conserved among its homologs in *Acinetobacter* species, including SA01-OmpA (Fig. 2a) (33). The predicted three-dimensional model confirmed that SA01-OmpA consists of a transmembrane β-barrel and a periplasmic domain (Fig. 2b). The OmpA β-barrel in SA01 was structurally superimposed with that of A. baumannii 17978 and revealed that the sequence of the β-strands is highly conserved. The most variable regions were typically observed in the loops that connect the strands of the β-barrel, the loop which links the β-barrel to the periplasmic domain, and the C terminus as the end of the protein (Fig. 2c).

### Gene cloning, heterologous expression, and emulsifying ability of SA01-OmpA.

**FIG 3:**
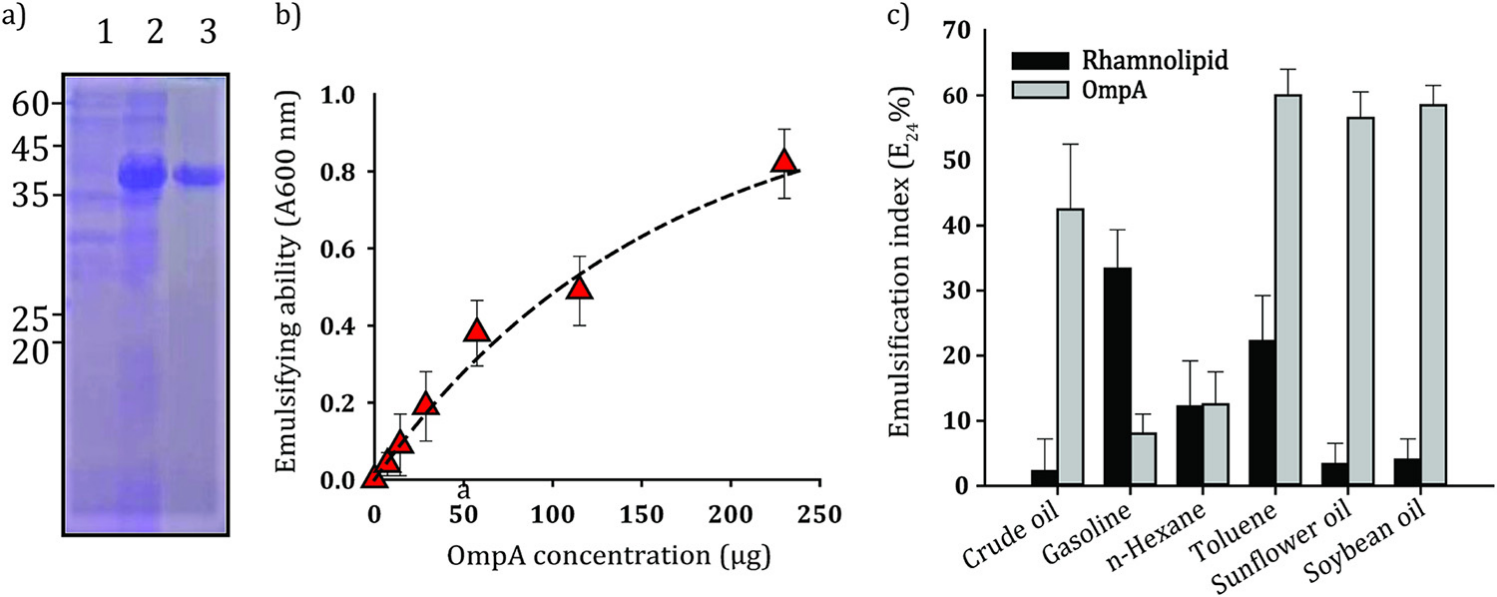
Purification and evaluation of the emulsifying capacity of purified recombinant OmpA protein. (a) SDS-PAGE analysis of recombinant OmpA. (1) Soluble protein fraction of the cells before induction. (2) Soluble protein fraction after 6 h induction. (3) His-tag-purified recombinant protein. (b) Emulsifying ability of different concentrations of OmpA using 2-methylnaphthalene and hexadecane (a control containing hydrocarbon alone was subtracted). (c) Emulsification index (E_24_) of OmpA and rhamnolipid using different vegetable oils and hydrocarbons.

The SDS-PAGE profile of the purified recombinant OmpA protein demonstrated a band with an approximate molecular weight of about 40 kDa (Fig. 3a). The purified recombinant OmpA was used to evaluate its emulsifying properties. The results showed that the emulsifying ability of the OmpA protein was proportional to its concentration, although the curve is not entirely linear (Fig. 3b). The specific emulsifying activity was 79 ± 8 U/mg, and the emulsions were stable over several weeks. The emulsifying capacity of recombinant OmpA and rhamnolipid biosurfactant, with an equal amount (150 μg), were compared to each other using six hydrophobic organic compounds (Fig. 3c). The recombinant OmpA achieved a significant emulsifying ability, especially the highest value of the emulsification index (E_24_), being about 60, 58, and 56.5% for toluene, soybean, and sunflower oil, respectively. The lowest values of the emulsification index obtained with gasoline and *n*-hexane were about 8.33 and 12%, respectively. The SA01-OmpA was able to emulsify crude oil with the E_24_ value of 42%. In comparison with rhamnolipid, OmpA demonstrated a higher emulsifying function when confronted with crude oil, sunflower oil, soybean oil, and toluene. However, it showed a weaker emulsification effect on gasoline. There was no significant difference between the emulsification index of OmpA and rhamnolipid from *n*-hexane.

### Identification of OmpA in intracellular and extracellular fractions.

**FIG 4:**
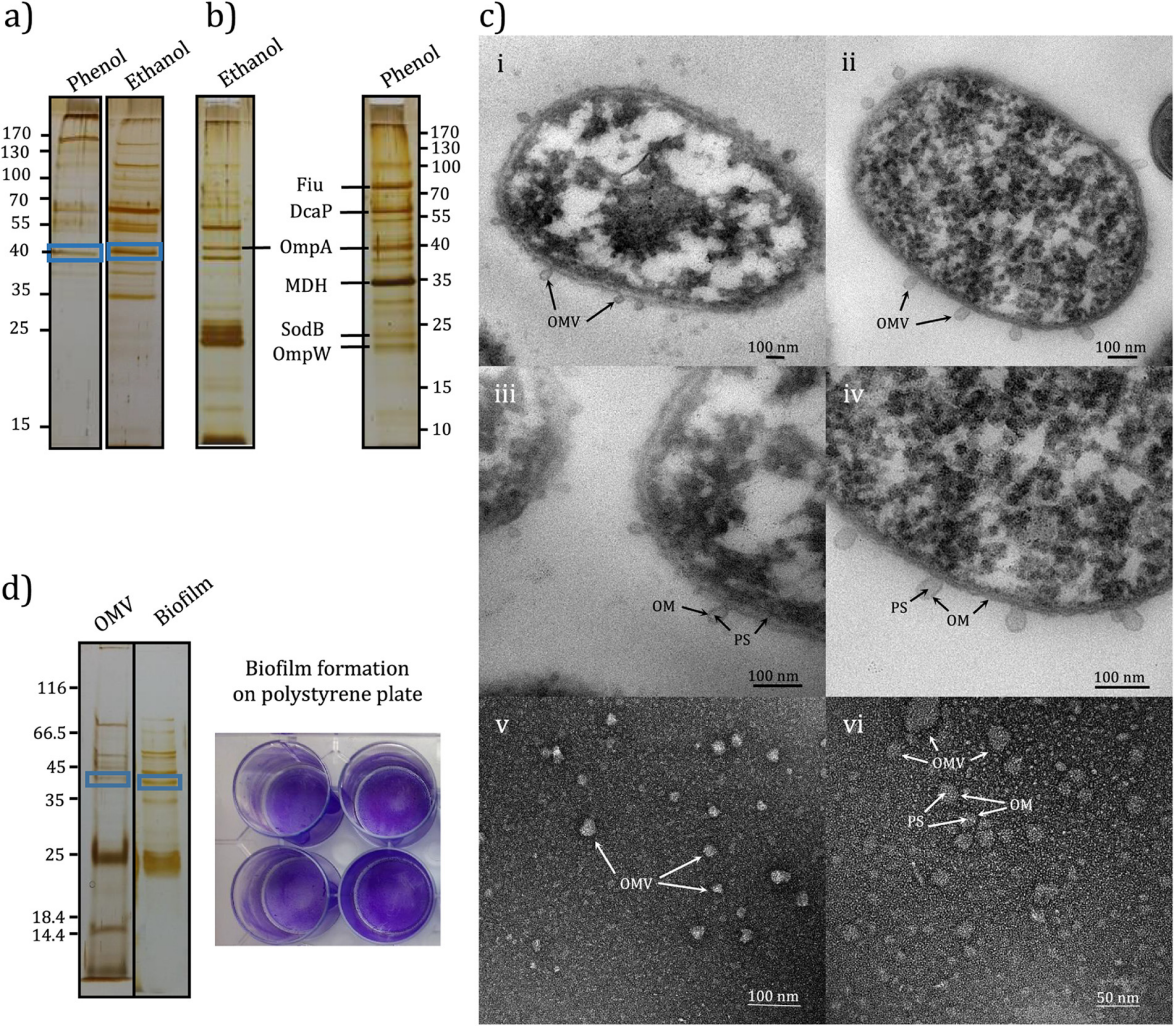
Identification of OmpA among different protein contents of cellular and subcellular fractions in *Acinetobacter* sp. SA01. (a) Identified OmpA in membrane protein SDS-PAGE profile of the cells fed with 1 g/liter phenol and 10 g/liter ethanol (enclosed by blue box). (b) Cell secretome as feeding with ethanol and phenol as the sole carbon source. Proteins (40 μg/ml) were analyzed and separated by SDS-PAGE (12.5%) and stained using silver-staining procedure. (c) Determination of OMV produced by strain SA01. (i and ii) Extrusion of OMVs from outer membrane. (iii and iv) Outer membrane (OM) and periplasmic space (PS). (v and vi) Precipitated OMVs from cell-free supernatant. (d) Bands affiliated with OmpA (in blue box). The protein profile of OMV was obtained from OMVs generated in cell-free supernatant of the cells grown in M9 minimal medium containing 1 g/liter phenol, shaking rate of 180 rpm (30°C), in late logarithmic phase. The protein profile of biofilm matrix was obtained by SA01 cells grown in the same medium as OMV was obtained but in a 12-well tissue culture plate incubated at 30°C with no shaking for 48 h.

Examination of the membrane fraction using silver-stained SDS-PAGE followed by nLC-MS/MS identified a band at ∼40 kDa as OmpA (Table 2) (Fig. 4a). A comparison of secretory proteins of the cells fed with 10 g/liter ethanol and 1 g/liter phenol by SDS-PAGE analysis revealed a visual change in their protein profiles. However, OmpA was detected in both conditions (Fig. 4b). We were able to identify OMV produced by the SA01 strain as an environmental isolate (Fig. 4c). Transmission electron microscopy (TEM) images have shown small spherical-shaped structures with an average size of 50 nm that forced out of the outer membrane. Like conventional OMVs, they are composed of a one-bilayer membrane that originated from the outer membrane of bacterial cells and surrounded the periplasmic contents. Considering the possibility of differences in OMV-associated proteins in different strains or the effect of altered conditions on its protein composition (34), we were able to detect OmpA among the OMV proteins of cells grown in the presence of 1 g/liter phenol (Fig. 4d). Shotgun proteomics analysis of OMV derived from *Acinetobacter* sp. SA01 exhibited that the proteins in OMV were not due to cell lysis (Data Sets S3 at https://doi.org/10.6084/m9.figshare.13415789). Outer membrane proteins such as OmpA and PIB (PorB) (major outer membrane protein), as well as proteins like SodB that are present in the periplasmic space (35), compared to catechol-1,2-dioxygenase and aconitate hydratase with high levels of expression in the cells, represented a higher relative abundance in the OMV protein profile. Therefore, according to our evidence, the presence of each of these proteins seems unlikely due to cell lysis. It was further confirmed through filtration of cell lysis by the Amicon 50-kDa cutoff spin filter, and results indicated that OmpA secreted out of the cell as OMV packages (Fig. S3 at https://doi.org/10.6084/m9.figshare.13415789). Further studies have shown that the SA01 strain, like A. baumannii strains, can form biofilm on polystyrene surfaces, and OmpA was among the proteins present in the biofilm matrix (Fig. 4d).

**TABLE 2:** OmpA identification using nLC-MS/MS

Parameter	Value
Locus tag (GenPept accession no.)	WP_166170865
No. of amino acids	351
Description	OmpA family protein
SEQUEST HT score	1,147.35
Sequence coverage (%)	50
No. of unique peptides	16
Sequenced peptides	1) [K].AAGSIADGESEEGTLGNAGLGAFWR.[L], 2) [K].DQYKPEIAK.[V], 3) [K].GDVEGLNVPAGAEYK.[Q], 4) [R].IEGHTDNTGPR.[A], 5) [K].IKPYVLLGAGHYK.[Y], 6) [R].LNDALSLR.[T], 7) [R].LSTQGFAWDQPIADNNTK.[E], 8) [K].LVEYPNATAR.[I], 9) [K].QTQINGNFYATSDLITK.[N], 10) [R].RVFATISGSR.[T], 11) [K].SNIKDQYKPEIAK.[V], 12) [K].SSLVNEYNVDPAR.[L], 13) [R].TVVAEPAQQAPAAQ.[-], 14) [R].VFATISGSR.[T], 15) [R].VFFDTNK.[S], 16) [R].VFFDTNKSNIK.[D]

### Kinetics of growth and pH variations during consumption of sodium acetate, ethanol, and phenol.

**FIG 5:**
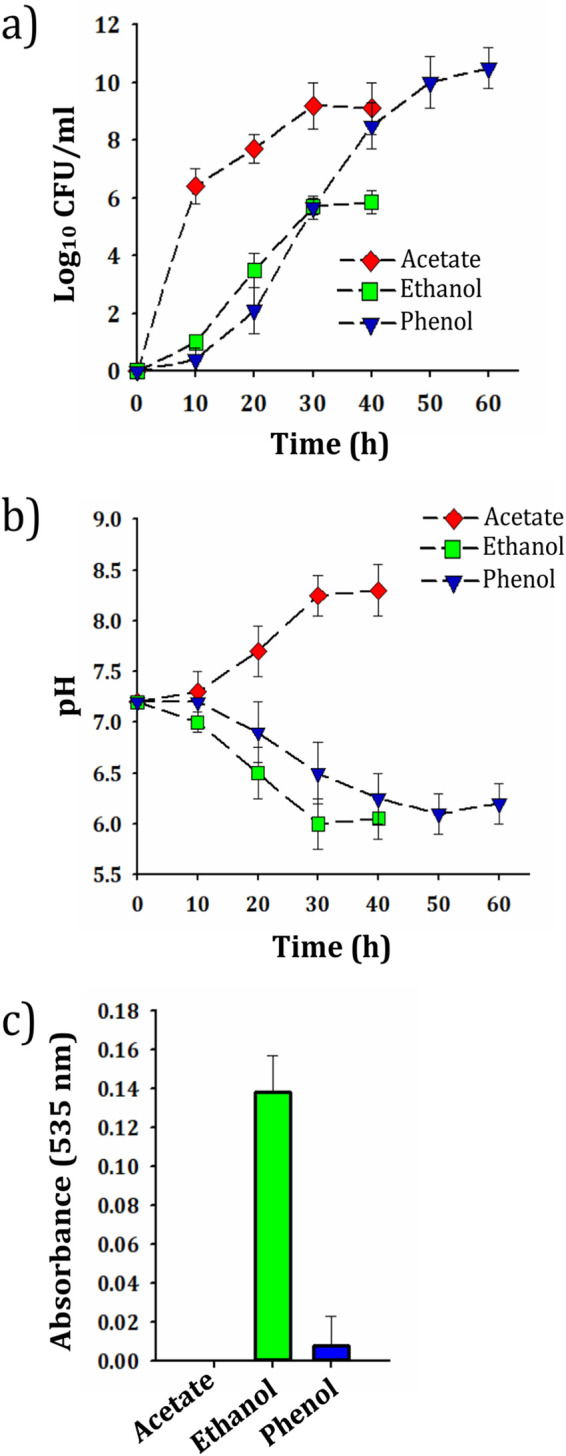
(a) Quantification of viable bacteria (CFU values) in culture medium containing phenol, ethanol, and sodium acetate. (b) Changes in culture medium pH by SA01 cells during consumption of sodium acetate, ethanol, and phenol. (c) Levels of IAA produced by SA01 cells grown on sodium acetate, ethanol, and phenol as they reach the early stationary phase of growth.

To evaluate whether the nature of the carbon source and induced stresses resulting from it affect the expression of OmpA, sodium acetate (1 g/liter), ethanol (10 g/liter), and phenol (1 g/liter) were used and added to the culture medium as the sole carbon source. Our studies have shown that in addition to phenol, the SA01 strain can grow in a medium containing ethanol and sodium acetate, consuming them as a carbon source (Fig. 5a). Low pH can increase oxidative stress, adversely affecting the accuracy of the data obtained (36). Thus, a further point to consider was the pH measurement of the culture medium (containing selected carbon sources), as the pH may be affected by the production of some by-products during carbon consumption. The results showed that the pH of phenol and ethanol-containing medium decreased over time (from 7.2 to ∼6.2 and 6, respectively), while the pH increased with sodium acetate consumption (from 7.2 to ∼8.3) (Fig. 5b). It has previously been shown that A. baumannii 17978 can produce indole-3-acetic acid (IAA) when grown in LB medium containing ethanol (21). Our results showed that IAA is significantly produced only in ethanol-grown cells, but despite the similarity in chemical structures of sodium acetate and acetic acid, it was not produced in the sodium acetate-containing medium (Fig. 5c).

### Evaluation of oxidative stress in phenol, ethanol, and sodium acetate-grown cells.

**FIG 6:**
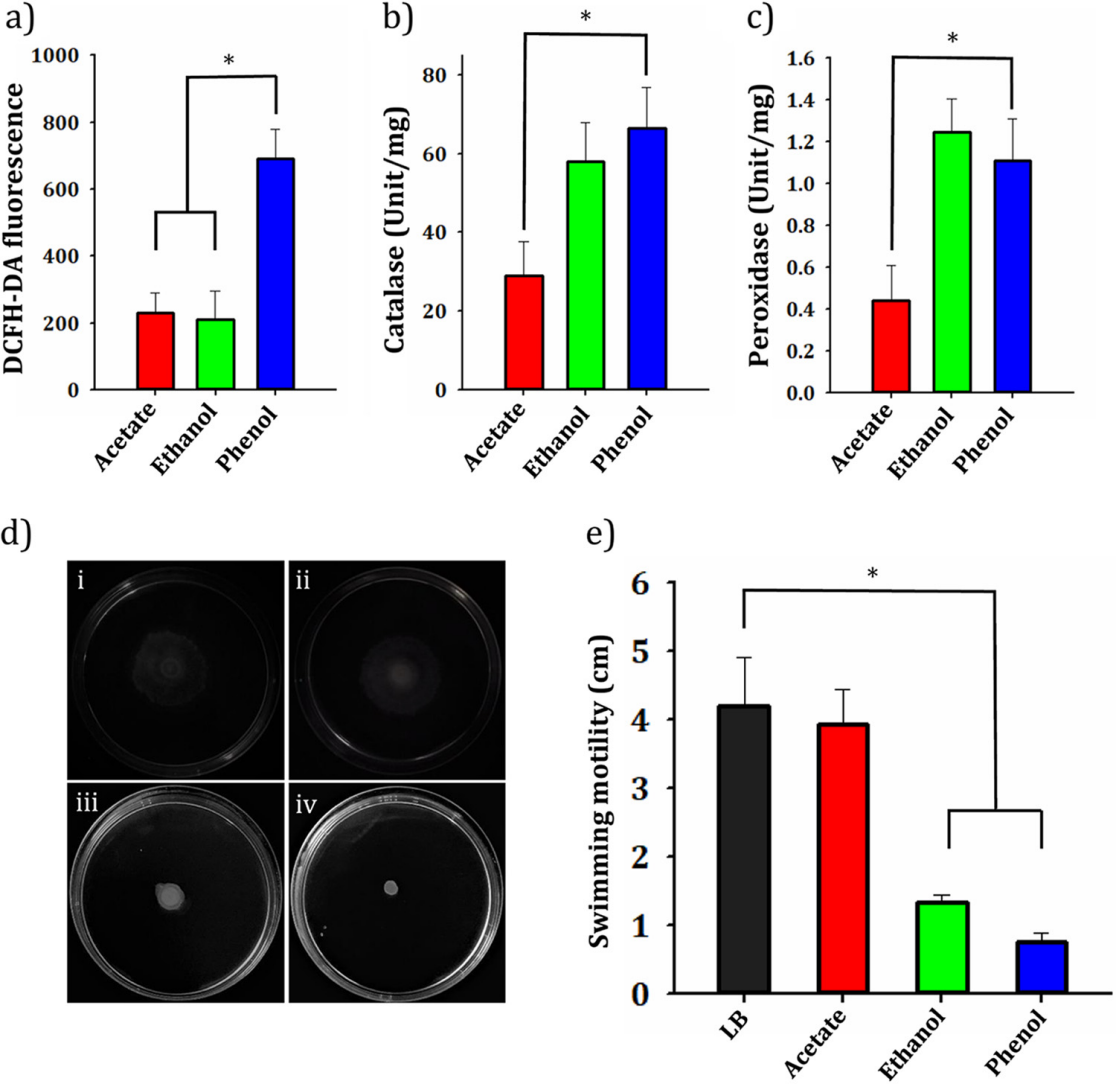
Effect of sodium acetate, ethanol, and phenol on oxidative stress levels and cellular motility of the SA01 strain. (a) Measurement of ROS levels using fluorescent probe 2′,7′-dichlorofluorescein diacetate (H_2_DCFDA). (b and c) Catalase and peroxidase activity of the cells grown on different carbon sources. (d) Cell motility assay with swim plate containing (i) no additional content (control), (ii) sodium acetate (1 g/liter), (iii) ethanol (10 g/liter), and (iv) phenol (1 g/liter). (e) Diameter of the radius produced by swimming motility of *Acinetobacter* sp. SA01 under given conditions. Asterisks above each bar represent significant difference (one-way ANOVA; Duncan test; *, *P < *0.05).

An attempt was made to compare the oxidative stress response of SA01 cells during assimilation of ethanol (10 g/liter) and phenol (1 g/liter) with sodium acetate-grown cells (1 g/liter). Since oxidative stress delineated as an imbalance between the production of reactive oxygen species (ROS) and antioxidants, the levels of both free radicals and antioxidants were evaluated (37). All samples were taken in the late exponential or early stationary phases of growth. The cellular redox state was measured by fluorescent dye 2,7-dichloro-dihydrofluorescein diacetate (DCFH_2_-DA). Concerning the alterations of antioxidant capacity in cells, the activity of the catalase and total peroxidase enzymes, as involved in the elimination of H_2_O_2_, were evaluated. The results showed that sodium acetate-fed bacterial cells had lower levels of ROS and H_2_O_2_-removing activity than cells fed with phenol (Fig. 6a to c). While cell growth in the presence of ethanol-like sodium acetate led to lower production of ROS, a higher catalase and peroxidase activity was observed to be about the same as that of phenol-fed cells. The swimming motility assay showed that SA01 cells grown on a semisolid plate containing 10 g/liter ethanol or 1 g/liter phenol had less mobility than sodium acetate (1 g/liter) and control subjects (Fig. 6d).

### OmpA expression in SA01 cells grown in medium containing phenol, ethanol, and sodium acetate.

**FIG 7:**
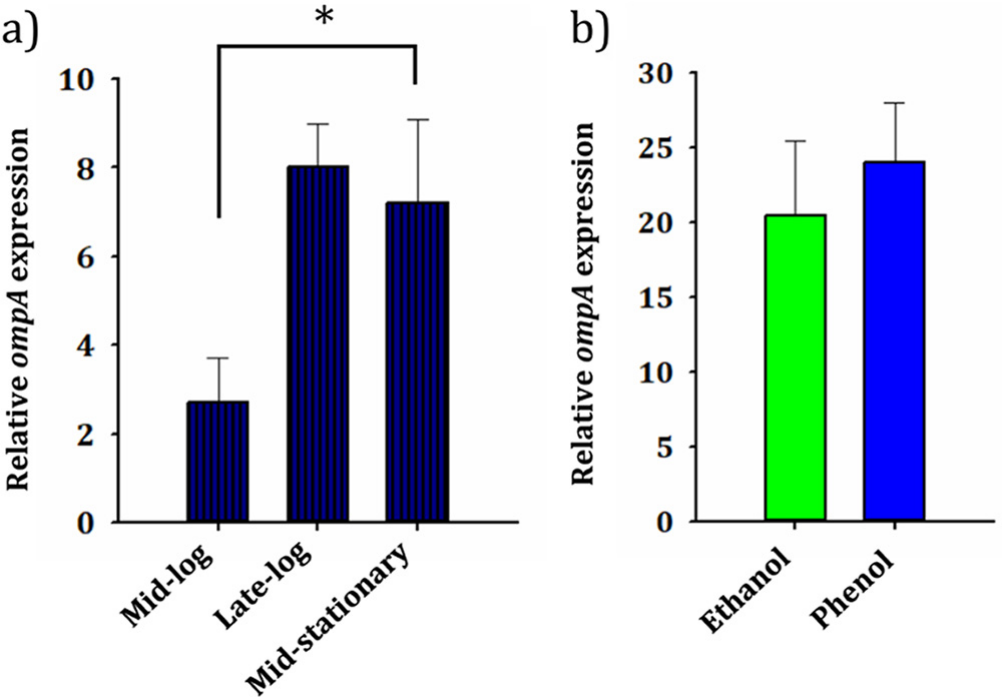
Relative expression levels of *ompA* gene under different growth conditions. (a) Pattern of growth phase-dependent gene expression of SA01 cells. The value of threshold cycle (*C_T_*), obtained from early logarithmic phase, was considered the reference. (b) Changes in *ompA* expression when fed with different carbon sources (sodium acetate, ethanol, and phenol). The value of *C_T_* obtained from sodium acetate was considered a reference. Asterisks above each bar represent significant difference (one-way ANOVA; Duncan test; *, *P < *0.01).

The protein composition of cells can change in different growth phases regarding physiological conditions. In this regard, a comparative analysis was carried out to evaluate the expression of OmpA in several distinct growth phases. Cells were cultured in 1 g/liter phenol, and the samples were taken from early log, mid-log, late log, and mid-stationary phases. The results indicated only a 2.7-fold increase in expression of OmpA at mid-log in comparison to early log phase, but up to 8- and 7-fold increases were observed at late log and mid-stationary phase, respectively (Fig. 7a). Therefore, samples were taken from the late log or early stationary phases to compare OmpA expression in cells fed with sodium acetate, phenol, and ethanol. The SA01 cells grown in medium containing phenol and ethanol showed 24- and 20-fold increases, respectively, in the expression of OmpA compared to sodium acetate-grown cells (Fig. 7b). However, there was no significant difference in the expression levels of OmpA between the cells grown in the presence of phenol and ethanol.

### Proteomic evaluation of cellular response to oxidative stress caused by phenol and ethanol.

**FIG 8:**
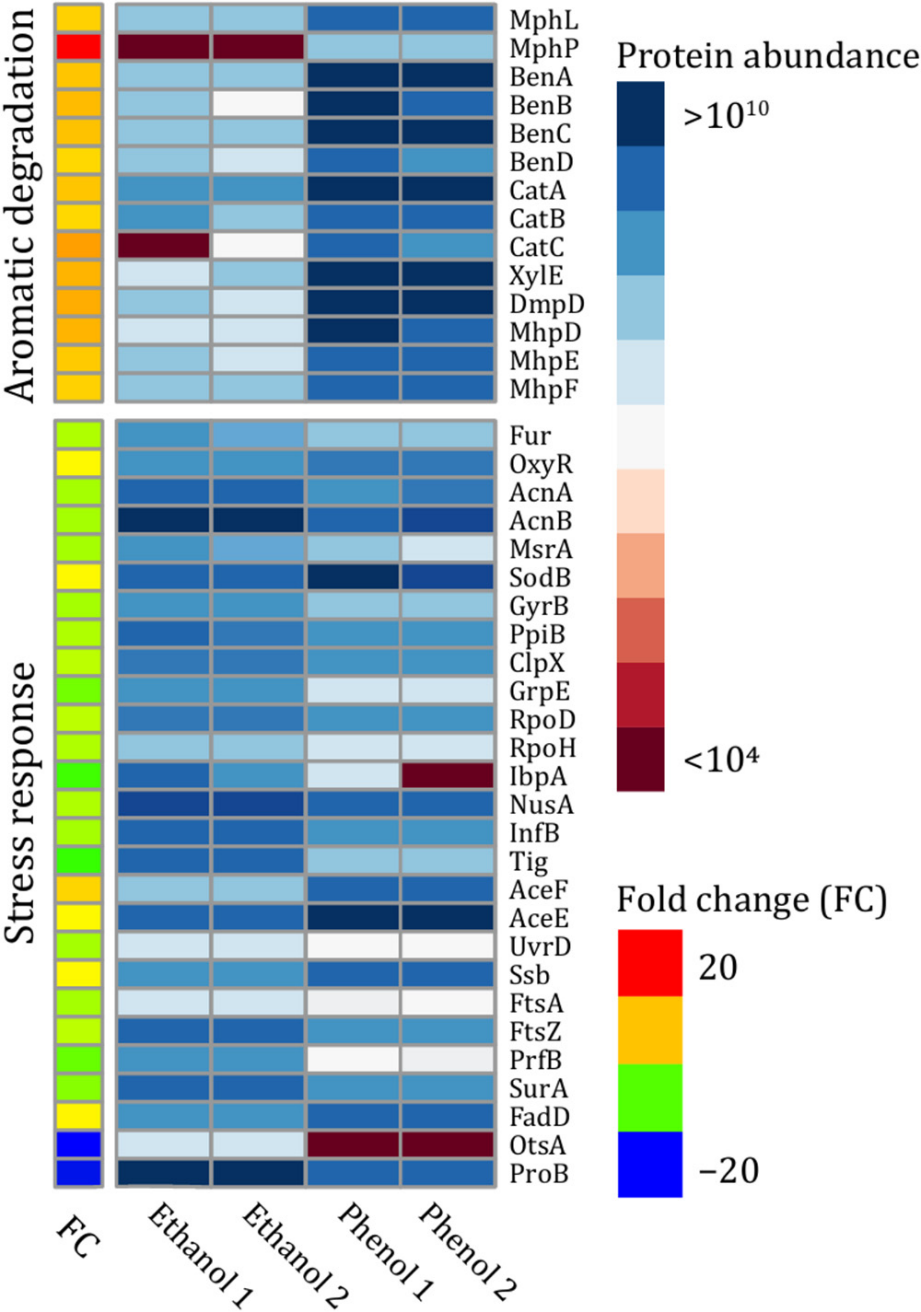
Differential expression of important proteins required for aromatic degradation and stress response of SA01 cells grown in two different carbon sources (phenol and ethanol).

Given the low level of ROS in ethanol-fed cells than in cells fed with phenol, the protein profile of SA01 cells exposed to 10 g/liter of ethanol and 1 g/liter of phenol was studied in more detail in response to oxidative stress. Dynamic proteome changes in the SA01 strain during ethanol and phenol consumption were evaluated using a *t* test with a *P* value of 0.05 and fold change of >1.5. A total of 295 proteins in these two conditions showed significant expression differences, of which 105 proteins in phenol and 190 proteins in ethanol-fed cells were upregulated (Data Sets S4 at https://doi.org/10.6084/m9.figshare.13415789). Previous computational analyses and transcriptome and ethanol-related genomic data showed multiple types of stress imposed on nonethanol-adapted E. coli cells, along with increased ROS levels (38). These include heat shock response, cold shock response, SOS response, envelope stress response, and general stress response. Here, we compared the expression levels of proteins in cells fed with phenol and ethanol to elaborate on the mechanisms underlying stress response for the two conditions used (Fig. 8). A slight increase (1.97-fold) in the expression of superoxide dismutase occurred in phenol-grown cells. However, despite the high expression of alkyl hydroperoxide reductase and catalase, there was no significant difference between the two conditions. Fur, an inhibitor of iron uptake protein with the potential of integrating into oxidative stress, showed a higher expression in ethanol-fed cells. More heat shock response proteins were overexpressed as a result of ethanol-induced stress. Meanwhile, ClpX and GrpE proteins with chaperone properties were 1.9- and 4.8-fold, respectively, and the RpoD and RpoH signal factors, capable of producing chaperones, proteases, and DNA repair enzymes, showed 2- and 2.5-fold increases in ethanol-grown cells, respectively. Concerning the cold shock response, the proteins NusA (DNA damage response protein), InfB (involved in the assembly/maturation of the ribosomal subunit), and Tig (chaperone protein) were overproduced in SA01 cells grown in the presence of ethanol. However, most of the proteins involved in central carbon metabolic pathways, also known as cold shock response protein, such as AceF, AceE, and Lpd, besides FadD, were overexpressed in SA01 cells fed with phenol. FtsA and FtsZ are among the most important proteins that showed a higher level of expression in the presence of ethanol than phenol, playing a crucial role in cellular processes such as cell division. The results also indicated that ethanol assimilation leads to a higher general stress response than those with phenol. Of these proteins, the most important is ProB, which was upregulated in ethanol-fed cells up to 22-fold. This protein contributes to a subpathway of proline biosynthesis, an essential amino acid involved in the stress tolerance of many organisms (39, 40). The outer membrane is directly exposed to the external environment in Gram-negative bacteria, serving as a protective barrier when faced with a change in environmental conditions. Porins have several significant functions, ranging from nutrient uptake to resistance to certain types of antibiotics. Therefore, the primary target of this study was OmpA protein. The analysis of shotgun proteomics data demonstrated that only DcaP, OprD, and NicP, among all known porins, are differentially expressed in SA01 cells grown in the presence of phenol, whereas no significant differences occurred for the expression of OmpA or other related proteins.

### Effect of other oxidative stress-inducing factors on OmpA expression.

**FIG 9:**
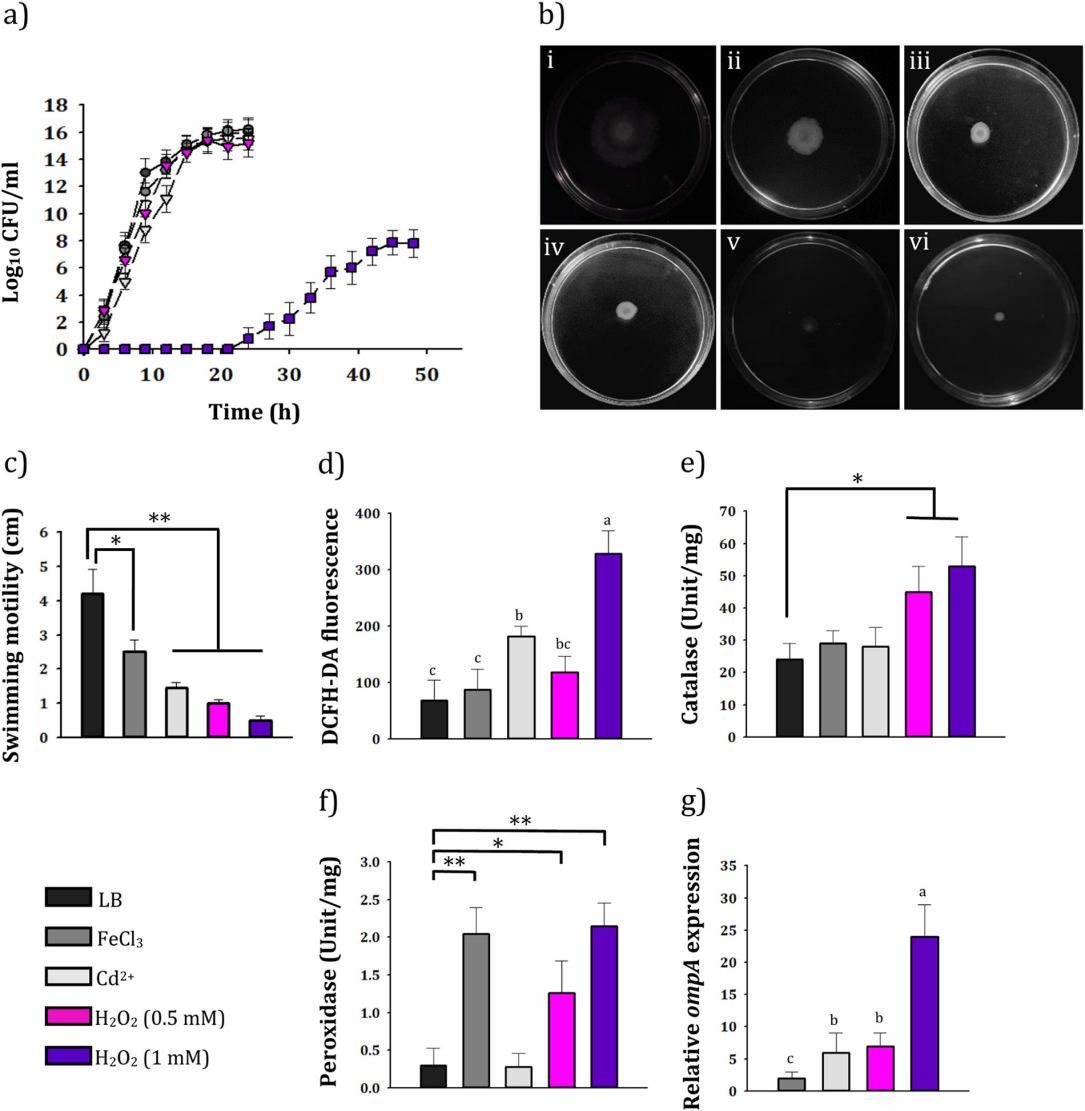
Assessment of the growth kinetics, oxidative stress, and comparative expression of OmpA under stressful conditions (100 μM FeCl_3_, gray; 10 μM Cd^2+^, white; 0.5 mM H_2_O_2_, pink; 1 mM H_2_O_2_, violet). Cells grown in LB medium without any additional content (black) were considered control. (a) Growth kinetics. (b) Motility assay with swim plates containing (i) no additional content (control), (ii) 100 μM FeCl_3_, (iii) 5 μM Cd^2+^, (iv) 10 μM Cd^2+^, (v) 0.5 mM H_2_O_2_, and (vi) 1 mM H_2_O_2_. (c) Diameter of the radius produced by swimming motility of *Acinetobacter* sp. SA01 under given conditions. Asterisks above each bar represent significant difference (one-way ANOVA; Duncan test; *, *P < *0.05; **, *P < *0.0005). (d) ROS level in cells. Means not sharing the same letter are significantly different (one-way ANOVA; Tukey test; *P < *0.05). (e and f) Catalase and peroxidase activity of the cells. Asterisks above each bar represent significant difference (one-way ANOVA; Duncan test; *, *P < *0.05; **, *P < *0.01). (g) Relative expression of *ompA* under stated conditions. The value of *C_T_*, obtained from control was considered the reference. Different letters above the bars indicate significant differences (one-way ANOVA; Tukey test; *P < *0.05).

To further evaluate the correlation between expression of OmpA and the level of oxidative stress, H_2_O_2_ and cadmium, as two oxidative stress-inducing agents with no nutritional value, were employed for a complementary evaluation (41–44). Previous studies have also shown that iron (another ROS-generating agent) has the potential to increase the expression of OmpA in A. baumannii ATCC 19606 (19). Therefore, the cells were grown under iron-rich conditions (100 μM), and those without it were used as the control subjects. The bacterial cells were able to grow at all concentrations tested, except at 2 mM H_2_O_2_ and 20 μM cadmium (Fig. 9a). The growth rates of cells exposed to H_2_O_2_ (0.5 mM), cadmium (5 and 10 μM), and iron (100 μM) were very close to those of cells cultured in LB alone, except for the 1 mM H_2_O_2_, which had a significant adverse impact on bacterial growth. Besides, when cells were grown in the presence of H_2_O_2_ (0.5 and 1 mM) and cadmium (5 and 10 μM), the cell motility was significantly reduced. The addition of iron (100 μM) decreased mobility, but it was lower than the test concentrations of cadmium and H_2_O_2_ (Fig. 9b).

The results obtained from oxidative stress analysis (Fig. 9c to e) showed a higher level of oxidative stress of any given samples (especially for the cells exposed to 1 mM H_2_O_2_) than the control subjects. The measurement of ROS in bacterial cells cultured in the presence of 1 mM H_2_O_2_ showed a higher level than 0.5 mM, as followed by a higher catalase and peroxidase activity. The treatment of bacterial cells with cadmium resulted in a higher ROS production than the control subject. However, it did not significantly differ in terms of catalase and peroxidase activity. This may be due to either increased expression of other antioxidants such as alkyl hydroperoxide reductase or possibly detoxification of metal by the production of metallothioneins. Also, the levels of ROS and catalase activity in cells exposed to 100 μM iron appeared to get close to the control subjects, but peroxidase activity was higher. Although high peroxidase activity can be indicative of cellular stress in iron-treated cells, it appears to be less toxic to SA01 cells than cadmium. Reverse transcriptase PCR (RT-PCR) analysis showed that the expression levels of OmpA increased in all treatments tested compared to the same untreated subjects (Fig. 9f). The bacterial cells exposed to 1 mM H_2_O_2_ showed the highest level of OmpA expression with an approximately a 24-fold increase, while exposure to 100 μM iron leads to the lowest increase in expression level only by up to 2-fold. Also, the cells treated with 0.5 mM H_2_O_2_ and 10 μM cadmium showed 7- and 6-fold increases in OmpA expression, respectively.

## DISCUSSION

The role of some outer membrane proteins in stress and antimicrobial resistance of *Acinetobacter* species is very important from chemical, biological, biophysical, and environmental viewpoints. On the other hand, some bacterial outer membrane proteins deserve to be evaluated as surface-active compounds due to their amphiphilic features (10, 45) with biotechnological, industrial, and clinical significance. Therefore, extensive efforts have been made to examine, in more detail, the biological, structural, and functional aspects of these proteins. In this study, we attempted to obtain more shreds of evidence about the function of an OmpA from a native phenol-degrading bacterial strain, *Acinetobacter* sp. SA01. To gain insight into the underlying processes which were generated by oxidative stress, the identification of proteins affected by ROS is of great importance. We therefore took advantage of this property as a clear steer for defining the relationship between OmpA expression and oxidative stress caused by ROS in cells exposed to different cell culture conditions, as a consolidated purpose.

Phylogenetic analysis of the protein annotated by referencing WP_166170865 (in *Acinetobacter* sp. SA01) indicated a close correlation with the earlier OmpA of well-known *Acinetobacter* strains (32, 33). The structure prediction and phylogenetic analysis of this protein revealed little difference in the structure of OmpA in various *Acinetobacter* species, most of which are related to loops and C-terminal residues of these proteins. The β-barrel domain of SA01-OmpA, as shared similarities with that of A. baumannii 17978, shows that both proteins have a cylindrical tube of the same size. Therefore, it can be speculated that SA01-OmpA functions as a slow porin due to the low diameter of the transmembrane domain. Also, the similarity of the OmpA-like domain (including Asp271 and Arg286) in both proteins demonstrates that SA01-OmpA can anchor in the PGN layer, consequently controlling the OMV production and its stability.

It has been shown that A. radioresistens KA53 has four hydrophobic regions that are not present in E. coli OmpA and are responsible for the emulsifying properties of this protein (33). Our studies have revealed that these regions are not only present in OmpA of SA01 strain but are also considerably conserved among other species such as A. baumannii. The emulsifying ability of the recombinant SA01-OmpA corroborates the assumption that this protein may have the emulsifying capacity in most *Acinetobacter* species. In comparison with chemically synthesized surface-active compounds, the biological ones are less toxic and more eco-compatible (46). Rhamnolipids produced by Pseudomonas aeruginosa are well-known biosurfactants with high application potentials (47). Most microbial surfactants are substrate specific, increasing the solubility of structurally distinct hydrocarbons and capable of producing emulsions at different rates using different hydrocarbons (48). A comparison between emulsifying potentials of SA01-OmpA and those of P. aeruginosa rhamnolipids, with an equal amount, revealed a possible prospect for its application in pharmaceutical and food-related industries.

OmpA is bound to PGN in the form of a monomer or dimer (49, 50). Moreover, the periplasmic domain of OmpA can interact with other proteins such as Braun’s lipoprotein (51) or the inner leaflet of the outer membrane (50) that may suggest the low probability of free secretion of OmpA into the extracellular space. The simultaneous identification of OmpA in the membrane and OMV may indicate that OmpA in the SA01 strain, like A. baumannii, first settled in the outer membrane and then transferred to the extracellular space through the OMV biogenesis process.

While the predicted size of SA01-OmpA after cleavage of the signal peptide is 35.8 kDa, either the posttranslational glycosylation (23) or an abnormal SDS-PAGE migration can affect the molecular weight of the protein (52).

In addition to OmpA, other outer membrane proteins, like DcaP, Fiu, OmpW, and some proteins present in the periplasmic space, like SodB, secreted out of the cells during OMV bulging (35). Many reports indicate that membrane vesicles in some bacteria carry various macromolecules such as proteins, RNA, and DNA, maintaining the structural integrity of the biofilm matrix or even protecting cells against stressful conditions (53). The presence of OmpA in the SA01 biofilm matrix, along with its amphipathic properties, may suggest a function similar to that of A. baumannii and its importance in adhesion and biofilm formation (16).

After determining the characteristics of OmpA and gaining assurance over protein selection, the importance of the nature of carbon source and oxidative stress were assessed using sodium acetate, ethanol, and phenol. Pessione et al. (22) showed that the pH of the environment is decreased during consumption of phenol by A. radioresistens S13, while an increase in pH occurred in acetate-grown cells. On the other hand, in another study, similar evidence was achieved when ethanol was consumed by A. baumannii ATCC 17978 (21). The authors attributed the decrease in pH to the presence of IAA and suggested that acetate accumulation, which occurs during ethanol metabolism, could lead to IAA production. Besides, it has been shown that the IAA plays a significant role in gene expression regulation, creating stress resistance, and producing biofilms that can help make resistance in cells under stress (54–56). As the low pH can induce oxidative stress in the cells, to avoid wrong results or misargumentation, we found it necessary to test the pH and IAA production by the SA01 strain. The lack of IAA production by the SA01 strain fed with sodium acetate may indicate the direct conversion of this molecule into acetyl-CoA. It might be argued by different modulation of IAA biosynthesis under the influence of environmental factors such as pH (57).

The low activity of catalase and peroxidase, together with low levels of ROS generation, in the cells fed with sodium acetate suggests that this compound is less toxic than ethanol and phenol. The motility of A. baumannii 17978 is reduced following stress induction by 1% and 2% ethanol on semisolid agarose plates, suggesting that the reduction of motility might be due to the production of more extracellular substances, as followed by the formation of more sessile cells and biofilms (21). The drastic reduction of SA01 motility, in the presence of phenol and ethanol, confirmed that these compounds make more stress that affects the cells than sodium acetate. Although the level of ROS in the cells fed with ethanol was at the same level as in sodium acetate, the high catalase and peroxidase activity may justify the accumulation of H_2_O_2_ inside the cells. The low level of ROS in ethanol-grown cells can be argued by the antioxidant property of the produced IAA (58). The changes between protein profile of SA01-OMV or secretome of ethanol- and phenol-fed cells could be due to the effect of different metabolisms of these two carbon sources and morphological or physiological changes, as well as different stress-induced responses during the growth process in batch culture (Fig. S4 at https://doi.org/10.6084/m9.figshare.13415789). The presence of OmpA under these two conditions shows the importance of this protein for the cells. The RT-PCR results revealed that the expression of SA01-OmpA in ethanol- and phenol-fed cells is higher than sodium acetate, as corroborated by the level of oxidative stress induction under these conditions. To further validate the results, a global proteomics analysis was performed for the examination of protein complexes from SA01 cells fed with ethanol and phenol. The results demonstrated that the response to stress is more or less subject to change. Most of these changes are affected by the different metabolic fates of ethanol and phenol in the carbon utilization pathway. For example, the aconitate hydratase, an enzyme involved in several metabolic pathways (such as carbon fixation), displays increased expression in ethanol-grown cells. Also, the lower expression of Fur protein, an inhibitor of iron uptake (59), or a higher expression of OprD (with a role in cell adaptation to iron and magnesium deficiency [60]) in phenol-grown cells could be a consequence of the high expression of proteins related to the phenol degradation pathway, such as phenol hydroxylase, 2-halobenzoate 1,2-dioxygenase, and benzoate 1,2-dioxygenase. As part of iron-sulfur proteins, the clusters function as electron transfer components (61–63). Porin-like proteins with structural roles, including NicP (nicotinate degradation protein P) and DcaP (most abundant outer membrane [OM] diffusion channel protein in pathogenic A. baumannii strains), are overexpressed in phenol-fed cells and possibly involved in the aerobic degradation pathways (64, 65). Despite the high abundance of the most important proteins involved in the alleviation of H_2_O_2_, such as AhpF, AhpC, KatG, and KatE in the cells fed with ethanol and phenol, no change in expression level of these proteins was observed either. The lack of noticeable change in the level of oxidative stress response proteins under ethanol and phenol conditions, along with the lack of differential changes in the expression of OmpA, suggest the potential effect of oxidative stress as one of the main factors contributing to the regulation of OmpA expression.

Based on the results and increased OmpA expression in the late logarithmic and stationary phase of growth, as accompanied by increased cellular stress (66), it seems that nutrient deficiency might not be the direct cause of this increase. Upregulation of OmpA using some of the oxidative stress-inducing agents, especially cadmium and H_2_O_2_ with lack of nutritional value, indicated that a cell requirement for a nutrient does not necessarily increase OmpA expression. The higher OmpA gene expression in the cells exposed to cadmium compared to iron, with lower oxidative stress, and the correlation between the increase of OmpA expression and H_2_O_2_ concentrations (from 0.5 to 1 mM) by the increased production of oxidative stress, suggests that this protein plays a crucial role in cell homeostasis and function at higher levels of oxidative stress.

The role of OmpA in the face of oxidative stress has been reported through a variety of mechanisms (67, 68). It seems that the outer membrane proteins, such as OmpC in *Salmonella*, with large pores and high permeability, are closed under oxidative stress conditions (68). On the other hand, OmpA, which provides a smaller pore with less permeability in the membrane, is unplugged by the oxidation of the disulfide bonds formed by two cysteine residues in the periplasmic domain, thereby controlling membrane permeability. The thiol group present in amino acid cysteine is a known target by oxidants and has antioxidant properties (69–71). These two cysteine residues in the periplasmic domain of OmpA are highly conserved in most Gram-negative bacteria, so they tend to interact with oxidant agents (72). However, OmpA found in *Acinetobacter* strains lacks both cysteine residues. The inability of recombinant SA01-OmpA in the reduction of 2,2-diphenyl-1-picrylhydrazyl (DPPH) confirmed that the protein does not have antioxidant properties, which could be due to the lack of cysteine residues (data not provided). Therefore, it seems likely that the expression of OmpA is increased once SA01 cells experience oxidative stress as a result of harsh conditions, such as exposure to organic solvents or heavy metals, to protect cells through its low permeability, OMV, and biofilm formation. The presence of resistance genes and regulatory or mobile genetic elements, in addition to diverse metabolic pathways, can influence their reflex responses to stress or even the expression of some target proteins such as OmpA (73–76). All things considered, the SA01 cells seem to be able to utilize the OmpA protein during its adaptive evolution under stress conditions and exhibits several advantages for being deployed in *Acinetobacter* spp. upon encountering exogenous and endogenous factors leading to ROS generation. Apart from a vast array of functions in the medical and clinical fields (as a virulence factor in biofilm formation, immunomodulation, and antibiotic resistance), *Acinetobacter* OmpA has shown emulsifying ability in some strains and, accordingly, may play a role in the biodegradation of hydrophobic compounds, including hydrocarbons (77). Given the results, it seems feasible to evolve the *Acinetobacter* sp. SA01 further, particularly for more OmpA production on different types of hydrophobic substances, by tuning expression of OmpA in response to changing environmental conditions.

## MATERIALS AND METHODS

### Genome sequencing, assembly, annotation, and phylogenetic analysis.

Whole-genome sequencing of *Acinetobacter* sp. SA01 carried out using Illumina HiSeq 2500 system, following the procedure published elsewhere (78). In brief, a 150-bp paired-end reads library was constructed from 1 mg of gDNA according to the manual provided by the Nextera DNA library preparation kit (Illumina, CA, USA). FastQC v011.3 and Trimmomatic 0.32 were employed for qualitative control and trimming of raw data. SPAdes v3.11.1 and Quast v3.2 were employed to respectively perform *de novo* assembly from different K-mer combinations and quality assessment of the assembly. The optimal selected k-mer combination has resulted in 68 scaffolds arranged by ABACAS using the closest reference genome. Finally, RAST (available at http://rast.nmpdr.org/) and Prokka v1.10 were employed for identification and genome annotation of the provided genome assembly. The closely related bacterial strains, as identified by phylogenetic analysis of *Acinetobacter* sp. SA01 genome data using PhyloSift software and Bowtie2 v2.3.0 aligner, confirmed the results by reading mapping to all available reference genomes of related species. Furthermore, a phylogenetic tree was reconstructed based on the aligned sequences of the total proteome of selected bacterial strains from each species (367 out of 2864 available genomes) using the composition vector method available in CVTree_V3.0 (http://tlife.fudan.edu.cn/cvtree/cvtree/). The phylogenetic analysis of proteins was performed by using MEGA X software (maximum-likelihood estimation with 1,000 bootstraps), after a multiple sequence alignment achieved by the MUSCLE program.

### Phylogeny and *in silico* primary, secondary, and tertiary structure analysis of OmpA in *Acinetobacter* sp. SA01.

Protein sequence alignment was performed using the T-Coffee webserver and sequence similarities, and secondary structure elements were assigned using ESPript 3.0 (79, 80). Secondary and tertiary structures were predicted using the RaptorX server (81). Signal peptides were excluded from protein query sequences. Glycosylation sites were predicted using the GlycoPP webserver (82).

### Gene cloning, heterologous expression, and emulsifying ability of OmpA.

An appropriate pair of primers (containing NdeI and XhoI restriction sites) was designed for amplification of loci newly defined in this study (signal peptide was excluded) (Table S1 at https://doi.org/10.6084/m9.figshare.13415789). Digested and amplified DNA fragments were cloned into pET 26b and transformed into E. coli strain BL21(DE3). Induction for higher expression was performed using 0.5 mM isopropyl-β-d-thiogalactopyranoside (IPTG) when the optical density at 600 nm (OD_600_) of bacterial culture reached ∼0.5 (at 20°C, 180 rpm). Cells were sonicated, and His-tagged proteins were purified from cleared lysate using affinity chromatography (Qiagen, Germany). The total protein content was determined using bicinchoninic acid (BCA) assay (Thermo Fisher Scientific, USA). For further validation, the purified OmpA protein was subjected to SDS-PAGE and identified by nanoscale liquid chromatography-tandem mass spectrometry (nLC-MS/MS). The defined concentration of the purified OmpA was transferred to a 10-ml glass tube containing a mixture of 20 mM Tris-HCl, pH 7.0, and 10 mM MgSO_4_ (in 1.5 ml total volume). Then, 200 μl of mixture solution containing 2-methylnaphthalene and hexadecane (1:1 vol/vol) was added to the abovementioned mixture and vortexed for 5 min at room temperature. The turbidity was measured at 600 nm using a spectrophotometer (Jena Bioanalytic, Jena, Germany). The emulsifying ability and emulsification index (E_24_) were determined according to the procedures described earlier (9, 83). The rhamnolipids biosurfactant was extracted from P. aeruginosa as a prior-described procedure (84).

### Growth conditions and experimental design.

To evaluate the effect of different carbon sources on the production of OmpA, a defined amount of carbon source (sodium acetate, ethanol, or phenol) was added to the M9 minimal medium as previously described (29). The cultures were incubated at 30°C and shacking rate of 180 rpm after inoculation of 10^7^ CFU/ml initial cell concentrations. We placed 1 μl of starter culture in mid-log phase directly on the center of a semisolid medium (composed of 10 g/liter tryptone, 5 g/liter NaCl, and 0.3% agarose) containing intended toxic compound to perform swimming motility assay. The plates were incubated for 16 h at 30°C.

### Biofilm and OMV preparation.

Bacterial cultures were centrifuged at 14,000 rpm for 30 min, and the supernatant was filtered using a 0.22-μm Millipore membrane filter (Merck, Darmstadt, Germany). Ultracentrifugation (Beckman Coulter Optima MAX-XP ultracentrifuge) was performed at 100,000 × *g* for 1 h at 4°C, after ammonium sulfate precipitation (45%). The precipitates were dialyzed and the OMV pellet resuspended using 50 mM phosphate-buffered saline (PBS), pH 7.4 (34). The biofilm formed in a polystyrene well was washed three times by 50 mM PBS, pH 7.4, scraped off with a sterile scalpel, and resuspended in 50 mM phosphate buffer (pH 7.4). Then, NaOH was added to the final concentration of 400 mM, and reactions were stirred for 3 h (4°C). The suspension was centrifuged at 16,000 rpm for 40 min (4°C), and the pellet was resuspended in 50 mM phosphate buffer (pH 7.4). The suspension was filtered using 0.22-μM-pore-size membrane and dialyzed against the same buffer overnight (4°C) (85).

### Sample preparation for shotgun proteomics.

Cultures were harvested by centrifugation at 5,000 rpm for 15 min at 4°C, and the pellets were treated with 0.5 mg/liter lysozyme for 15 min on an ice bath. Afterward, the cells were disrupted using a sequential freeze-thaw cycle, and the cell debris, as well as intact cells, were eliminated by centrifugation (14,000 rpm, 30 min, and 4°C). The supernatant was carefully separated and used as total cellular protein for shotgun proteomics. The quantity of the proteins was determined using the BCA protein assay kit using the manual provided by the manufacturer.

### Extraction of secreted proteins from SA01 cells.

Bacterial cultures were centrifuged at 14,000 rpm for 30 min at 4°C, and the supernatant was passed through a 0.22-μM-pore-size membrane filter (Merck Millipore, MA, USA). To obtain total secretome, 1 ml of supernatant was picked up and lyophilized using SpeedVac vacuum concentrator (Thermo Fisher Scientific, MA, USA). Proteins were analyzed and separated by SDS-PAGE (12.5%) and stained using the silver-staining procedure. The selected protein bands were excised from gel using a sterile scalpel.

### Mass spectrometric analysis.

The procedure was performed according to a previously reported protocol by Seidel et al. (86). For in-gel protein digestion, the gel slices were destained using appropriate protocol for the silver-stained proteins, followed by cysteine derivatization and tryptic in-gel digestion. Desalting was performed using zip-tip C18 pipette tips provided by Merck Millipore. Then, the peptide samples were applied in an Orbitrap Fusion Tribrid mass spectrometer (Thermo Fisher Scientific), coupled with a nano-LC system (Dionex Ultimate 3000 rapid separation liquid chromatography [RSLC]; Thermo Fisher Scientific) and equipped with an Acclaim PepMap100 C18 LC column (Thermo Fisher Scientific).

### Total RNA extraction and RT-PCR studies.

Total RNA was extracted using GeneAll RiboEx Total RNA kit (Seoul, South Korea) using the protocol provided by the manufacturer after harvesting cells. After assessing the quality and quantity of the extracted RNA, DNase treatment was performed using DNase I, RNase-free kit (Thermo Fisher Scientific, MA, USA) to remove genomic DNA from the transcriptome. Afterward, the cDNA was synthesized from 0.5 μg of DNase-treated total RNA using a quick guide provided by the BioFact Company (Daejeon, South Korea).

The pair of primers was constructed based on the sequence of the *ompA* (target gene) and 16S rRNA (housekeeping gene) in *Acinetobacter* sp. SA01 (Table S1). Sanger sequencing confirmed the authentication of the intended amplicon provided by the primer sets. The primer efficiency was determined using different dilutions of primers, and control with no reverse transcriptase was used to confirm the lack of contaminating DNA. Real-time DNA amplification was performed using SYBR green-based fluorescence assay, and RealQ Plus 2× Master Mix Green (Amplicon, Odense, Denmark) was used for the setup of the reaction in a real-time thermal cycler, taking the following steps: 5 min initialization at 95°C and 40 cycles of denaturing, annealing, and extension at the respective temperatures of 95°C, 58°C, and 72°C (20 min for each step). Finally, the relative gene expression was calculated using the relative expression software tool (REST 2009).

### Cellular ROS measurement.

Oxidative species were detected using an assay based on DCFH_2_-DA dye as previously described elsewhere (87). In brief, cultures were harvested by centrifugation at 4,000 rpm for 20 min at 4°C. The pellets were washed with 50 mM phosphate buffer (pH 7.4). Suspensions were adjusted to a final OD_600_ of ∼1, and DCFH_2_-DA was added to give a final concentration of 200 nM. The samples were washed using 50 mM PBS (pH 7.4) after 45 min incubation at room temperature. The intensity of fluorescence was assessed at excitation of 480 nm and emission of 520 nm using the Cary Eclipse fluorescence spectrophotometer (Mulgrave, Australia).

### Determination of activity of intracellular antioxidant enzymes.

The harvested cells were resuspended in 50 mM phosphate buffer (pH 7.4) and sonicated. The total extracted proteins from clear supernatant were quantified using BCA assay and employed for the measurement of enzyme activity. The intracellular proteins at the final concentration of 70 μg/ml were added into the 50 mM phosphate buffer (pH 7.4) containing 25 mM H_2_O_2_ to start the reaction, measuring catalase activity. The change in absorbance was tracked at 240 nm to monitor the consumption of H_2_O_2_ (88). A test was set up based on the Trinder's glucose activity assay to measure the peroxidase activity (89). The crude protein, at the final concentration of 70 μg/ml, was added to 50 mM phosphate buffer (pH 7.4), containing 1 μM phenol, 0.01 μM 4-aminoantipyrine, and 150 mM H_2_O_2_ to perform the experiment. Peroxidase activity was measured by tracking the quinone color at 508 nm.

### Indole-3-acetic acid production assay.

Cultures from the early stationary phase were centrifuged at top speed, and the supernatant was vortexed with Salkowski reagent (12 g/liter FeCl_3_ in 7.9 M H_2_SO_4_) at a 1:1 mixing ratio. The absorbance was measured at 535 nm after incubation of mixtures at room temperature for 30 min (21).

### Transmission electron microscopy.

The cells were centrifuged at 4,000 rpm for 15 min at 4°C and washed with 50 mM PBS, pH 7.4, three times. Samples were prepared as previously described by Alidoust et al. (88). The sections were placed on copper grids and coated with carbon for TEM (Philips CM 200) observation.

### Statistical analysis.

One-way analysis of variance (ANOVA) with *post hoc* test (Duncan or Tukey), based on the assumption of homogeneity of variance, was used to determine statistically different groups via SPSS (v22.0) software. For proteomic data analysis, two sample *t* tests with Benjamini-Hochberg false discovery rate (FDR) were performed via Rstudio to determine the changes of the two groups.

### Data availability.

Information about the genome assembly of *Acinetobacter* sp. SA01 is available at http://www.ncbi.nlm.nih.gov with BioProject accession no. PRJNA599585. The mass spectrometry proteomics data have been deposited to the ProteomeXchange Consortium via the PRIDE partner repository (90) with the data set identifier PXD017288.
